# Collision Avoidance With Multiple Walkers: Sequential or Simultaneous Interactions?

**DOI:** 10.3389/fpsyg.2018.02354

**Published:** 2018-11-30

**Authors:** Laurentius Antonius Meerhoff, Julien Pettré, Sean Dean Lynch, Armel Crétual, Anne-Hélène Olivier

**Affiliations:** ^1^Inria, Univ Rennes, CNRS, IRISA - UMR 6074, Rennes, France; ^2^Univ Rennes, Inria, M2S - EA 7470, Rennes, France

**Keywords:** locomotion, multiple interactions, collision avoidance, dynamic gap, interpersonal coordination, affordance, perception-action, pass-ability

## Abstract

Collision avoidance between multiple walkers, such as pedestrians in a crowd, is based on a reciprocal coupling between the walkers with a continuous loop between perception and action. Such interpersonal coordination has previously been studied in the case of dyadic locomotor interactions. However, when walking through a crowd of people, collision avoidance is not restricted to dyadic interactions. We examined how dyadic avoidance (1 vs. 1) compared to triadic avoidance (1 vs. 2). Additionally, we examined how the dynamics of a passable gap between two walkers affected locomotor interactions. To this end, we manipulated the starting formation of two walkers that formed a potentially pass-able gap for the other walker. We analyzed the interactions in terms of the evolution over time of the Minimal Predicted Distance and the Dynamics of the Gap, which both provide information about what action is afforded (i.e., passing in front/behind and the pass-ability of the gap). Results showed that some triadic interactions invited for sequential interactions, resulting in avoidance strategies comparable with dyadic interactions. However, some formations resulted in simultaneous interactions where the dynamics of the pass-ability of the gap revealed that the coordination strategy emerged over time through the bi-directional interactions between all walkers. Future work should address which circumstances invite for simultaneous and which for sequential interactions between multiple walkers. This study contributed toward understanding how collision is avoided between multiple walkers at the level of the local interactions.

## Introduction

In a crowd, interactions between people at the micro-level construe how the crowd moves at the macro-level (Vicsek and Zafeiris, 2012). From a movement science perspective, these interactions are a form of interpersonal coordination: the coordination of one's movements with one (or more) other(s) (Schmidt and Richardson, 2008). An important aspect of interpersonal coordination in a crowd is regulating one's distance with others. Distance regulation requires a continuous coupling between perception and action of all persons involved, as each persons' actions affect - to some extent - the actions of others. Numerous studies have addressed distance regulation between two persons (i.e., dyads) with regards to interception (e.g., Passos et al., 2008; Zhao and Warren, 2017), and following/tracking (Meerhoff and de Poel, 2014; Meerhoff et al., 2014, 2017; Rio et al., 2014). Moreover, using an orthogonal avoidance task, multiple studies have addressed how dyadic interactions unfold through bidirectional interactions (Olivier et al., 2012, 2013; Basili et al., 2013; Huber et al., 2014; Lynch et al., 2017). When extrapolating these findings to distance regulation with more than two people (e.g., walking through a crowd of people), the multiple interactions make it more complex to study how human behavior emerges (Davids et al., 2014). An extensive literature on pedestrian crowds exists. Some studies adopt a macro-level approach disregarding the micro-level interactions (e.g., Degond et al., 2013). These macro-level approaches are highly informative to predict how crowds behave, however, the perception-action loops that underlie these macro-level patterns cannot be specifically studied. In contrast, microscopic approaches focus on local interactions (Paris et al., 2007; Van den Berg et al., 2008); however, in simulations arbitrary rules are set to combine multiple interactions, as the way humans deal with this is unexplored. Therefore, we examined how dyadic (between two persons) and triadic (between three persons) interactions compare in an orthogonal avoidance task.

The coordination of pedestrians at the level of local interactions can be described using an affordance-based approach (e.g., Fajen, 2007). An affordance is an opportunity for action that is furnished by the environment to an agent (i.e., an entity with decision-making ability such as a pedestrian). Gibson (1979) emphasized that affordances simultaneously depend on the agent's action boundaries and the configuration of the environment. For example, it has been shown that humans can perceive the pass-ability of a gap as a ratio of the width of the gap and their shoulders (Warren and Whang, 1987; Wilmut and Barnett, 2010; Franchak et al., 2012; Hackney et al., 2015). In other words, these affordances cannot be attributed to either the agent or the environment but must be considered in the agent-environment system (Warren, 2006). A pedestrian in a crowd usually does not come close to its action boundaries, however, the environment - cluttered with other pedestrians - may indeed present a strong limitation on what behavior is afforded. For a walker to interact with this environment, the interactions with the other pedestrians determine for the most part what behavior is afforded. By describing the relation between two pedestrians, Olivier et al. (2012, 2013) developed a measure that describes this agent-environment system. They quantified the time-evolution of the Minimal Predicted Distance (*MPD*), which is the linearly extrapolated predicted minimal interpersonal distance (i.e., the future distance of closest approach assuming a constant speed and heading). Although *MPD* is not a perceptual variable, it is an apt descriptor of the action afforded to either walker. A high enough *MPD* affords passage without collision. By comparing *MPD* at the end of an interaction with the start, Olivier et al. (2012) showed that walkers consistently adapted their trajectories when the risk of collision was high enough (i.e., initial *MPD* < ~1 m). Hence, an *MPD* below this threshold did not afford a collision-free passage and thus required some form of adaptation. Moreover, the temporal evolution of *MPD* indicates that collision is avoided proactively, with distinct observation, reaction and regulation phases (Olivier et al., 2013). Such proactive control has been put forward as one of the characteristics of an affordance (Fajen et al., 2009). Therefore, we adopt similar metrics that describe the agent-environment system with a strong emphasis on the other agent(s) in the environment.

Locomotor trajectories toward a target have been described as a stereotyped behavior in terms of path geometry and velocity profile (Hicheur et al., 2007), indicating that some generic principles may govern trajectory generation. However, when an obstacle is in motion, collision avoidance may need to be controlled on-line, as the behavior afforded in relation to moving objects may change over time (Cutting et al., 1995; Plumert and Kearney, 2014). Cinelli et al. (2008) showed that when passing through a moving aperture, participants used a perceptual feedback mechanism to assess whether collision could be avoided by solely changing speed or that shoulder rotations were required as well. Moreover, interpersonal coordination has a strong social component (Schmidt et al., 2011); for example, humans also regulate distance to preserve personal space (Bailenson et al., 2003; Gérin-Lajoie et al., 2005). Additionally, humans reciprocally influence each other, but not necessarily symmetrically (Meerhoff and de Poel, 2014). Therefore, it is important to study collision avoidance behavior in the context of human-to-human interactions. Nevertheless, dyadic (i.e., pairwise) pedestrian interactions show robust regularities in terms of adaptation thresholds (Olivier et al., 2012). Furthermore, these dyadic interactions often take place without inversion of crossing order, that is, the walker that was predicted (based on a linear extrapolation) to cross first at the start was most likely to indeed cross first at the end of the interaction (Olivier et al., 2013; Knorr et al., 2016). It can thus be surmised that although avoiding collision with other people requires a more adaptive strategy compared to avoiding static obstacles, these reciprocal interactions follow some clear regularities.

Some of the characteristics of dyadic interactions may be extrapolated to situations where many pedestrians interact (e.g., a crowd). However, when multiple persons coordinate their movements, the complexity rapidly increases, as each person can potentially interact with each other person (and vice versa). This has previously been described in interactive sports (e.g., Davids et al., 2014; Passos et al., 2016), and specific joint-action tasks (e.g., Richardson et al., 2015). It raises the question whether collision avoidance between many pedestrians can be described as a sequence of many dyadic interactions, or as one simultaneous interaction. One of the few studies (Dicks et al., 2016) that compared pedestrian interactions between two and three walkers, examined the potential for social interaction during a pedestrian crossing. In their study, the potential for social interaction was manipulated by having the oncoming walkers cross with or without looking at a mobile phone. Results revealed that the potential for interaction decreased the velocity, perhaps because the predictability is increased when somebody is looking at their phone. Additionally, the authors noted that participants took longer to complete a crossing with two compared to only one oncoming pedestrian. However, it was beyond the scope of their study to tease apart how these interactions differed. In this paper, we therefore aim to contrast the principles that govern dyadic and triadic interactions in a collision avoidance task.

Using an affordance-based approach (Fajen, 2007), the interactions between many pedestrians can be considered as a collection of gaps that may afford either passing through, or going around (Fajen et al., 2009). In traffic, such gaps have been studied extensively (e.g., Chihak et al., 2010; Louveton et al., 2012; Plumert and Kearney, 2014). For example, Louveton et al. (2012) suggested that drivers interact with the gap that exists between two cars when crossing a busy interaction. The action that is afforded can be described as the “pass-ability” of the dynamic gap that exists between these cars (Plumert and Kearney, 2014). Chihak et al. (2010) found evidence for a multistage interception strategy when passing through a moving gap on a bicycle. Rather than changing and maintaining heading and speed to shift the point of constant bearing to the desired point of intersection, participants consistently accelerated between 4 and 6 s before the passing through the gap. That is, initially participants decelerate (more than strictly necessary to cross behind the first object) and subsequently accelerate to safely pass through the gap. It could be argued that the invitation to act upon the affordance of passing through the gap only becomes apparent as the interaction unfolds (Withagen et al., 2017). Although pedestrian interactions are different from interactions in traffic (because of the imposed traffic rules and the different velocities -and thus “costs” of collisions), the notion of online control and emerging affordances is highly relevant for pedestrian interactions. This was for example highlighted by Cinelli et al. (2008), who put forward that walkers pass through a moving door (cf., dynamic gap) by controlling their trajectories on-line to constantly adjust to changing affordances. By describing the interactions in terms of their affordances, it becomes apparent that monotonic control laws may not entirely explain how trajectories emerge. In this paper, we thus also set out to quantify which behavior is afforded in relation to the gap that may exist between two persons.

**Figure 1:**
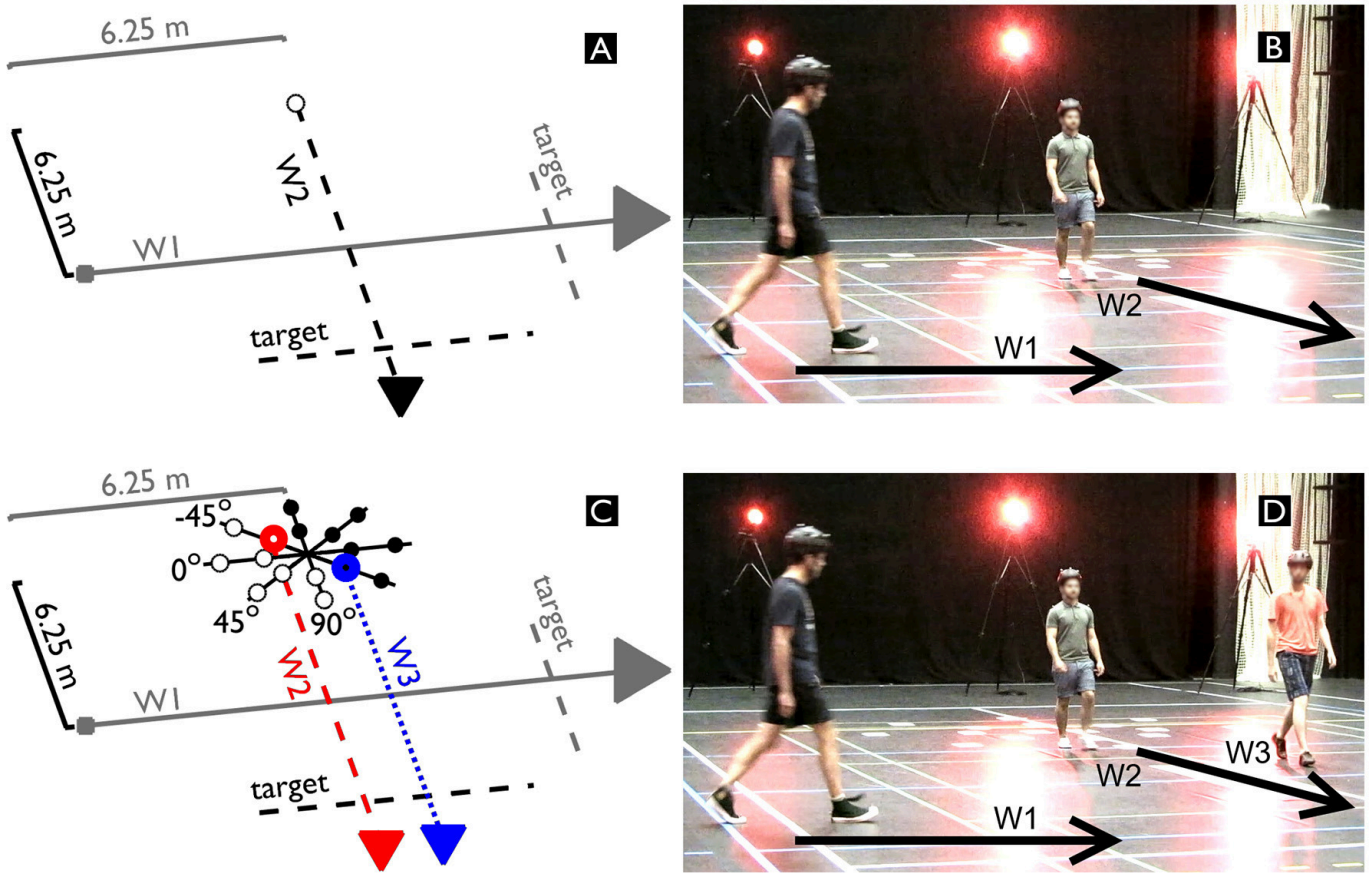
Schematic displays **(A, C)** and photos **(B, D)** of a dyadic **(A, B)** and triadic **(C, D)** trial during which each participant had to reach the target line on the other side of the interaction area. Walker 1 (W1) always started at 6.25 m from the center of the interaction area. In the dyadic trials, Walker 2 (W2) also started at 6.25 m. In the triadic trials, the center of W2 and Walker 3 (W3) was set at 6.25 m. The formation of W2 and W3 was angled at −45°, 0°, 45°, or 90°, with a diameter of 2 or 4 m. The participants provided written informed consent for the publication of these images.

Human movement follows regularities at various levels (cf., law-like principles, Turvey, 1990). Schmidt et al. (1990), for example, argued that patterns from within-person coordination (Kelso, 1984), may also apply to between-person coordination (e.g., Harrison and Richardson, 2009; Riley et al., 2011; Meerhoff et al., 2014). Following the same reasoning, we examine whether the principles from dyadic interactions (Olivier et al., 2012, 2013; Basili et al., 2013) may also apply to triadic interactions, as a step toward understanding the micro-level interactions in crowds of pedestrians. Therefore, the aim of this study was to examine how dyadic and triadic interactions compare. First, we examined whether similar initial parameters in terms of *MPD* yielded similar changes in *MPD* over time in triadic compared to dyadic interactions. We hypothesized that triadic interactions evoke a simultaneous interaction, therefore yielding different changes to similar initial parameters. Second, we examined whether the hypothesized simultaneous adaptation affected how often the crossing order inverted. We hypothesized that in the triadic interactions these role inversions are more frequent compared to dyadic interactions, as the strategy simultaneously depends on multiple persons. Additionally, we explored whether we can describe triadic interactions in a measure that quantifies the action-opportunities that are afforded to the walkers. To this end, we manipulated the relative starting formation of the group, which formed a potentially pass-able gap for the other walker (see Figure 1). With two pedestrians crossing the trajectory of another, there are three actions afforded to the single pedestrian: (1) in front of the other two, (2) through the gap between the other two, or (3) behind the other two. As an extension of *MPD*, we adopted the measure Dynamic Gap (*DG*) that described the pass-ability of the gap at each point in time. We hypothesized that the initial parameters of the triadic interaction in terms of *DG* better predict the outcome compared to the *MPD*.

In short, we addressed three research questions: (1) Are triadic interactions similar to two subsequent dyadic interactions (i.e., sequential treatment)? (2) Does a triadic interaction yield a rigorous adaptation subverting the starting parameters of each of the two dyadic interactions in a triadic interaction? (3) Can the avoidance strategy in a triadic interaction be explained in terms of a dynamic gap? We considered interactions to be treated sequentially when *MPD* was similar in the dyadic and triadic trials both at the start and at the end of the interaction. Subsequently, we assessed whether the simultaneous interaction strategy can be captured using *DG*. We hypothesized that the same consistencies in dyadic interactions do not transfer to triadic interactions, as multiple persons may be interacted with simultaneously. As an alternative we provided a triadic variable that provides additional insight as to how multiple persons are interacted with.

## Methods

### Participants and apparatus

Twelve participants (8 males and 4 females, aged 30 ± 7 years) volunteered to take part in this experiment. The participants were recruited through general advertisement within the research institute and local notice boards. The participants were randomly allocated to four groups of three participants. Two groups consisted of males only (aged 34 ± 11 and 32 ± 8 years), one group consisted of females only (aged 30 ± 7.5 years) and one group was mixed with 2 males and 1 female (aged 29 ± 5 years). Participants typically did not know each other before the experiment; however, this was not recorded. All participants had normal or corrected-to-normal vision and no known motor impairments that affected their walking ability. This study was carried out in accordance with the recommendations of the research institute. All participants gave their written informed consent in accordance with the declaration of Helsinki. The experiment took place in a motion capture laboratory with an 18 camera VICON motion capture system (Oxford Metrics Group Ltd., Oxford, UK) covering an interaction area of 13.5 × 17.5 m. Participants' trajectories were recorded with a sampling rate of 120 Hz using a retro-reflexive marker on each shoulder, the center of which was used to represent the participants' displacement. Additionally, we used a set of markers on a helmet to identify each participant.

### Procedure

Figure 1 provides an overview of the experiment showing the dyadic (Figures 1A,B) and triadic trials (Figures 1C,D). For each of the four experimental sessions, we recruited three participants to fulfill the roles of Walker 1 (W1), Walker 2 (W2), and Walker 3 (W3). W2 and W3 formed a group and crossed perpendicular to W1. We instructed all walkers to “reach the target line on the opposite side of the interaction area.” We did not provide any additional information as to how they were to do so. In the dyadic interactions (see Figures 1A,B), both W1 and W2 started at 6.25 m from the center of the interaction area. In the triadic interactions (see Figures 1C,D), the starting positions of W2 and W3 were varied in relative angle (−45°, 0°, 45°, or 90°) and radius (2 or 4 m), yielding eight different formations providing a range of starting parameters that would change the characteristics of the gap between W2 and W3. The relative position of W2 and W3 was symmetrical as such that the center between W2 and W3 was fixed at 6.25 m. Theoretically, this implied that without any adaptation of any of the walkers (i.e., constant speed and perfectly straight trajectories), W1 is precisely in the center of the gap between W2 and W3 (both equally far, but in opposite direction) after 6.25 m.

Each of the three participants of a group of participants performed each role (W1, W2, or W3) in every possible configuration, yielding 6 role configurations. For reasons of time, we performed all trials for one role configuration consecutively. The order of the configurations was randomized, and for each configuration all trials were presented in 3 randomized blocks. In the first block of 8 trials, participants performed each of 8 triadic formations crossing from a random side (i.e., to the left or to the right of W1). In the second block, W1 performed two dyadic trials with both remaining walkers in random order. In the third block, each of the 8 triadic formations was repeated, crossing from the opposite side as the first block. Once all 18 trials of a role configuration were completed, the participants were assigned new roles and the process was repeated. In total, we recorded 432 trials: 96 triadic trials (8 formations, 6 role configurations and 2 sides) and 12 dyadic trials (3 role configurations, 2 sides and 2 repetitions) for each of the four experimental sessions. No data points for any included trials needed to be interpolated. One trial (formation [90°, 4 m]) was excluded due to an unexpected technical malfunction, leaving 431 trials for further analysis. For each triadic formation and the dyadic formation, we analyzed 48 trials, except for the one missing trial in formation [90°, 4 m].

### Timing

**Figure 2:**
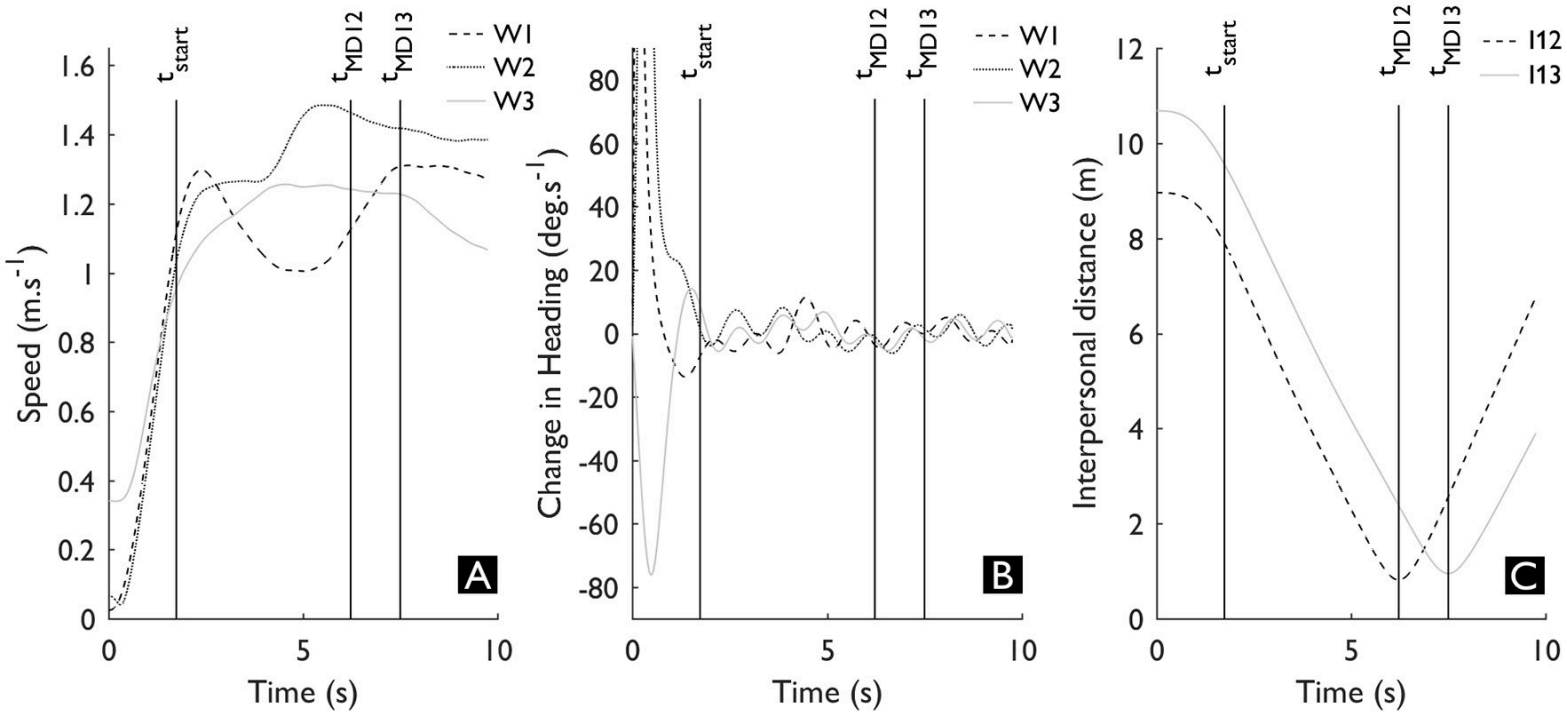
An exemplar trial showing the speed **(A)**, change in heading **(B)** of each walker, and interpersonal distances **(C)** between W1 & W2 and between W1 & W3. The speed (A) during a trial was used to identify t_start_, which corresponds to the instant the variable initiation phase was finished **(B)**. The interpersonal distances **(C)** were used to derive t_MD_.

We normalized the time-series from t_start_ until t_end_. Although of course a walker can already make adjustments during the acceleration phase, we are only interested in the adaptation made once a walker has reached a stable velocity. Therefore, we identified t_start_ as the first instant that any of the walkers had reached 90% of its maximum speed during that trial (see Figure 2A), which coincides with the highly variable instants at the start of a trial (as illustrated by the rate of change in heading in Figure 2B). Note that for each trial t_start_ was the same for all walkers during that trial. Next, we determined t_end_ of each interaction as the instant the minimal interpersonal distance between W1 and the other walkers occurred (t_MD12_ and t_MD13_, see Figure 2C). The trial duration was consistent (mean ± SD = 4.50 ± 0.06 s), but to allow for a direct comparison between trials we normalized time from t_start_ (0%) until t_end_ (100%).

### Kinematic analysis

We first post-processed the raw kinematic data. The medio-lateral sway movements that occur during gait were removed with a 3rd order low-pass Butterworth filter (cut-off frequency = 0.5 Hz). All kinematic analyses were performed in MATLAB R2015b (The MathWorks Inc., Natick, MA, 2015)^1^. We expressed specific interactions based on the walkers involved; we labeled the interaction between W1 and W2 as I12 and the interaction between W1 and W3 as I13. Additionally, any outcome variable that has the subscript “12” or “13” specifically refers to I12 or I13, respectively. We addressed the research questions using three different timeseries variables. Each outcome measure is explained below in the context of the specific research question.

#### Initial parameters

To assess whether similar initial parameters yielded similar avoidance strategies in triadic compared to dyadic interactions, we computed the Minimal Predicted Distance (*MPD* in meters) to their respective interactions (*MPD*_12_ and *MPD*_13_). This future distance of closest approach is a linear extrapolation of two walkers' current speed and heading to determine at which interpersonal distance they are predicted to cross assuming constant heading and speed (for more details, see Olivier et al., 2012, 2013). We tested the difference between the dyadic and triadic formations with Statistical Parametric Mapping (SPM; Pataky, 2010), which makes a two-tailed paired comparison at every time step^2^. We separately compared each of the eight triadic formations with the dyadic trials. To account for these multiple comparisons, we applied a Bonferroni correction to the critical *p-*value (*p* < 0.05). The corrected critical *p*-value was therefore set at *p* < 0.00625. When a significant effect was found during a trial, we reported the *t*-statistic corresponding to the maximum difference during that trial. Whenever a triadic formation had a similar *MPD*(t_start_) to the dyadic trials, a direct comparison was meaningful as the starting parameters were similar. If *MPD*(t_start_) was similar, but *MPD*(t_end_) different, this was considered evidence that the triadic interactions were not simply a summation of sequential dyadic interactions.

#### Crossing order inversions

**Figure 3:**
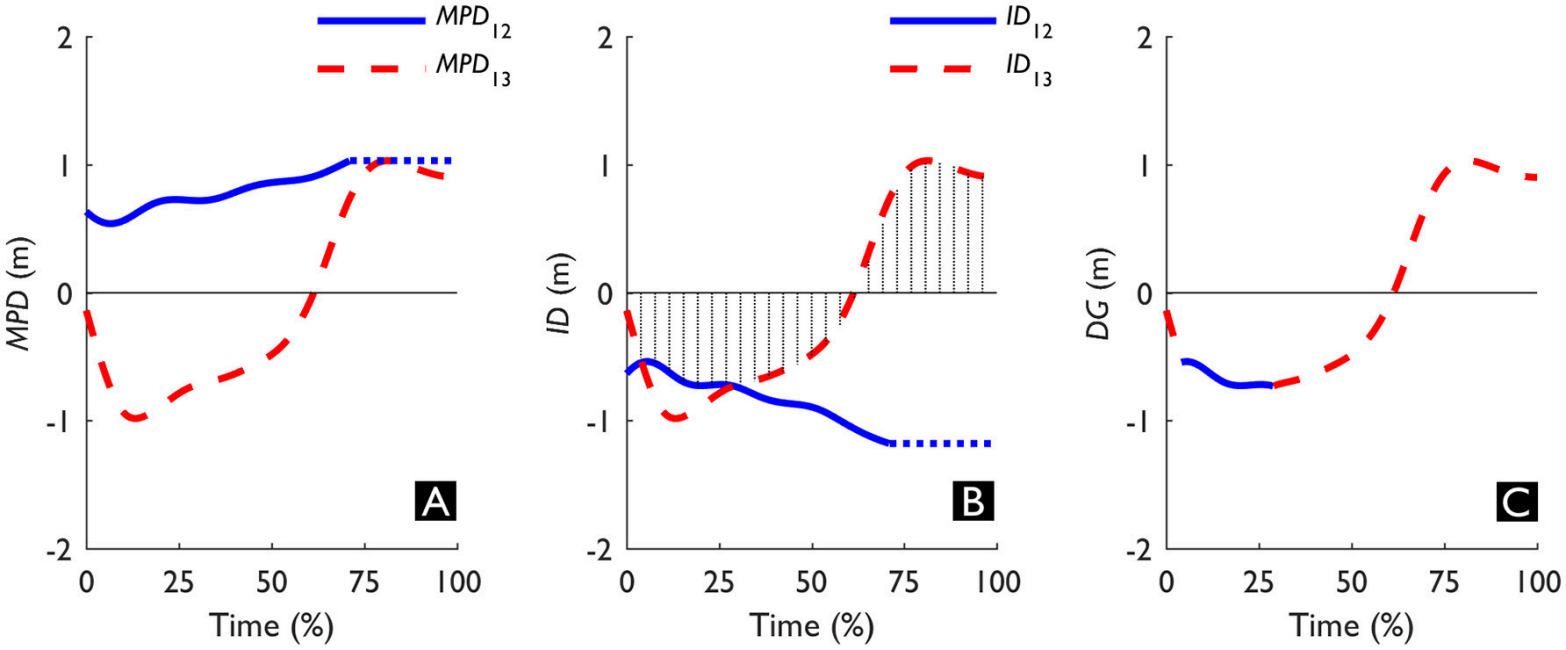
Exemplar data of one triadic trial showing the difference between **(A)** the Minimal Predicted Distance (*MPD*), **(B)** Interaction Distance (*ID*), and **(C)** Dynamic Gap (*DG*). The solid and dashed line refer to the interactions between Walker 1 (W1) & Walker 2 (W2) (i.e., interaction between W1 and W2, I12), and between W1 and W3 (i.e., interaction between W1 and W3, I13), respectively. The vertical lines in **(B)** indicate which interaction had the smallest absolute *ID* and upon which *DG* was based. The dotted horizontal line extending I12 in **(A, B)** denotes that the specific values were no longer updated as the time of minimal distance (t_MD12_) had already passed. See also Video 1 in the Supplementary Material for an animated display.

To further test whether triadic interactions are engaged simultaneously, we assessed whether the crossing order (i.e., W1 crossing first or second) changed more often in the triadic compared to the dyadic trials. Based on the same assumptions that we make to compute *MPD* (i.e., constant speed and heading), the crossing order can be computed by estimating who will first reach the point where the two trajectories are predicted to cross. The crossing order can be easily represented with *MPD* by assigning a positive sign to *MPD*(t_end_), and whenever during a trial the crossing order was predicted to be different compared to the crossing order at t_end_, the sign of *MPD* was negative. In the exemplar trial in Figure 3A, the negative *MPD* values of I13 (red dashed line) indicate that the predicted crossing order of W1 in relation to W3 was the opposite of the final crossing order from start (0%) until when the inversion occurred (62%). The predicted crossing order in I12 (blue solid line) on the other hand was the same throughout the whole trial as indicated by the consistently positive values. We reported the number of trials during which an inversion of crossing order occurred. To quantify the effect of formation on crossing in front, through or behind W2 and W3, we used a χ^2^ test (*p* < 0.05). If inversions occurred relatively more often in the triadic compared to the dyadic trials, it was considered evidence that W1 avoided collision with W2 and W3 simultaneously.

#### Gap pass-ability

**Figure 4:**
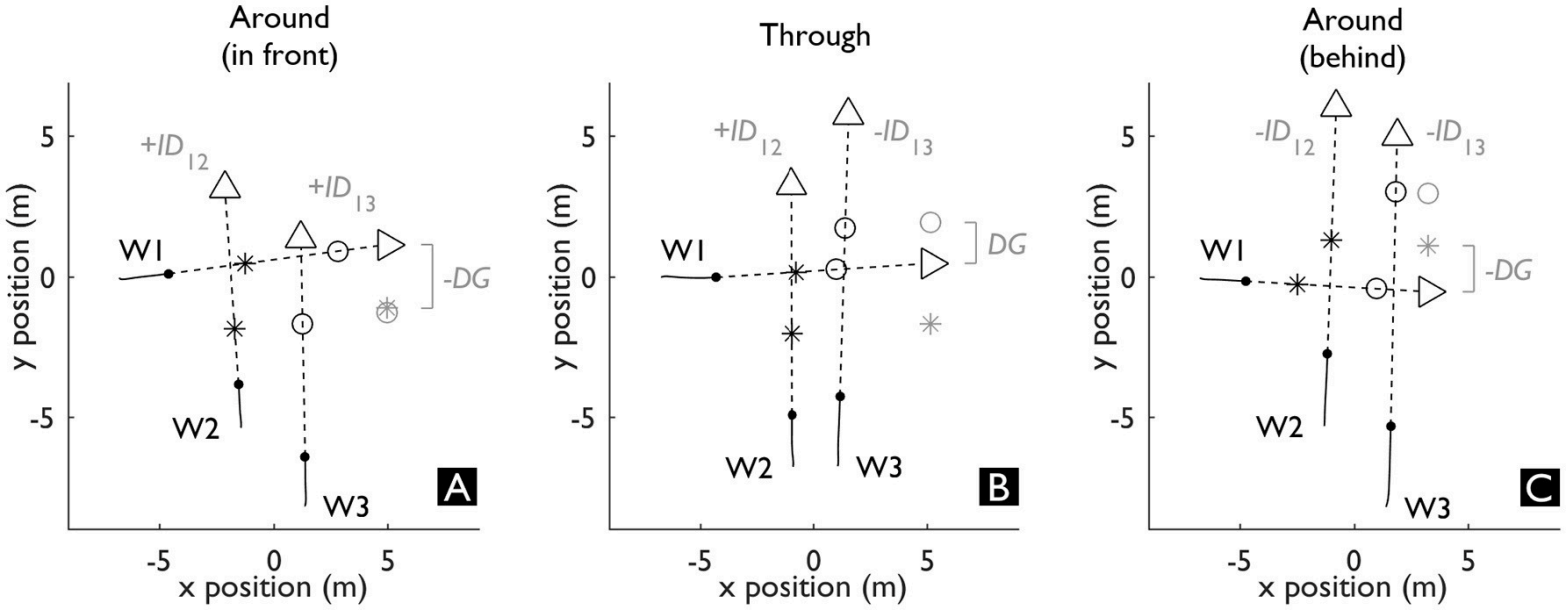
Positioning of all walkers in three exemplar triadic interactions where W1 was predicted to cross in front **(A)**, through **(B)** and behind **(C)** the gap. The • indicates the current position of each walker. The solid lines up until • depict the trajectory up until the current point in time. The dashed lines from • denote the predicted trajectories upon which the future positions of each walker are visualized at the instant of minimal distance: t_MD12_ (_*_) and t_MD13_ (◦). The distance between each ^*^ and ◦ corresponds with the *ID*, which is projected on the right in each panel as a portal that Walker 1 (W1) is predicted to pass through **(B)** or not **(A, C)**. *DG* is the distance to the closest side of the projected portal.

As an alternative to treating triadic interactions as a sequential summation of dyadic interactions, we explored whether we can describe triadic interactions with a measure that quantifies the action-opportunities that are afforded to W1 by simultaneously avoiding W2 and W3. To this end, we computed the Dynamic Gap (*DG*, see Figure 3C). *DG* is a combination of the Interaction Distances of I12 and I13 (*ID*_12_ and *ID*_13_, see Figure 3B), a derivative of *MPD*_12_ and *MPD*_13_. This derivative has the same magnitude as the *MPD*, however, the sign was based on the predicted crossing order at every time point in relation to W1: if W1 was predicted to cross first, *ID* was positive; if W1 was predicted to cross second *ID* was negative. Together, the signs of *ID*_12_ and *ID*_13_ could then be used to determine the predicted state (i.e., open or closed) of the gap that may exist for W1 between W2 and W3. If both *ID*s have the same sign, W1 is predicted to go around the gap (*ID*s > 0 represent W1 in front, see Figure 4A; *ID*s < 0 represent W1 behind, see Figure 4C) and the gap is thus closed. On the other hand, if the *ID*s have the opposite sign, it means that W1 is predicted to cross in front of one (i.e., positive *ID*) and behind the other (i.e., negative *ID*) walker and the gap is thus open (see Figure 4B). The interaction with the smallest absolute *ID* is then the interaction that constrains the state of the gap, as it represents the minimum adaptation required to change the state of the gap. This margin, in turn, can specify whether the gap affords passing through. Therefore, we determined *DG* as the smallest absolute *ID* and signed it based on whether W1 was predicted to go through (*DG* > 0 m) or around (*DG* < 0 m) the gap between W2 and W3. Note that the size of the gap between W2 and W3 as at least twice the magnitude of *DG*, as *DG* represents the smallest distance to one side of the gap.

In the exemplar trial in Figure 3B, given that *ID*_12_ was positive and *ID*_13_ negative at 100%, it can be deduced that W1 passed in front of W2 and behind W3. In Figure 3C, the negative *DG* indicates that until 62% the gap was predicted to be closed. However, from that point onward *DG* was positive, meaning that W1 eventually crossed between W2 and W3. To assess whether our manipulation of the formation yielded a broad range of behaviors, we examined whether formation affected the gap crossing behavior using a χ^2^ test (*p* < 0.05). Then, we compared the relative occurrence of inversions of *DG* in trials where W1 went through the gap with trials where W1 went around the gap using a χ^2^ test (*p* < 0.05). Using SPM, we performed six pairwise comparisons of *DG*, making each possible comparison between Open and Closed trials, with and without inversion. Again, we applied a Bonferroni correction to the critical *p-*value (*p* < 0.05) to account for the multiple comparisons. The corrected critical *p*-value was therefore set at *p* < 0.0083. The relative frequency of inversions in combination with when the difference between Open and Closed trials provides a description of how well this triadic measure (*DG*) describes the avoidance strategy.

## Results

### Initial parameters MPD

**Figure 5:**
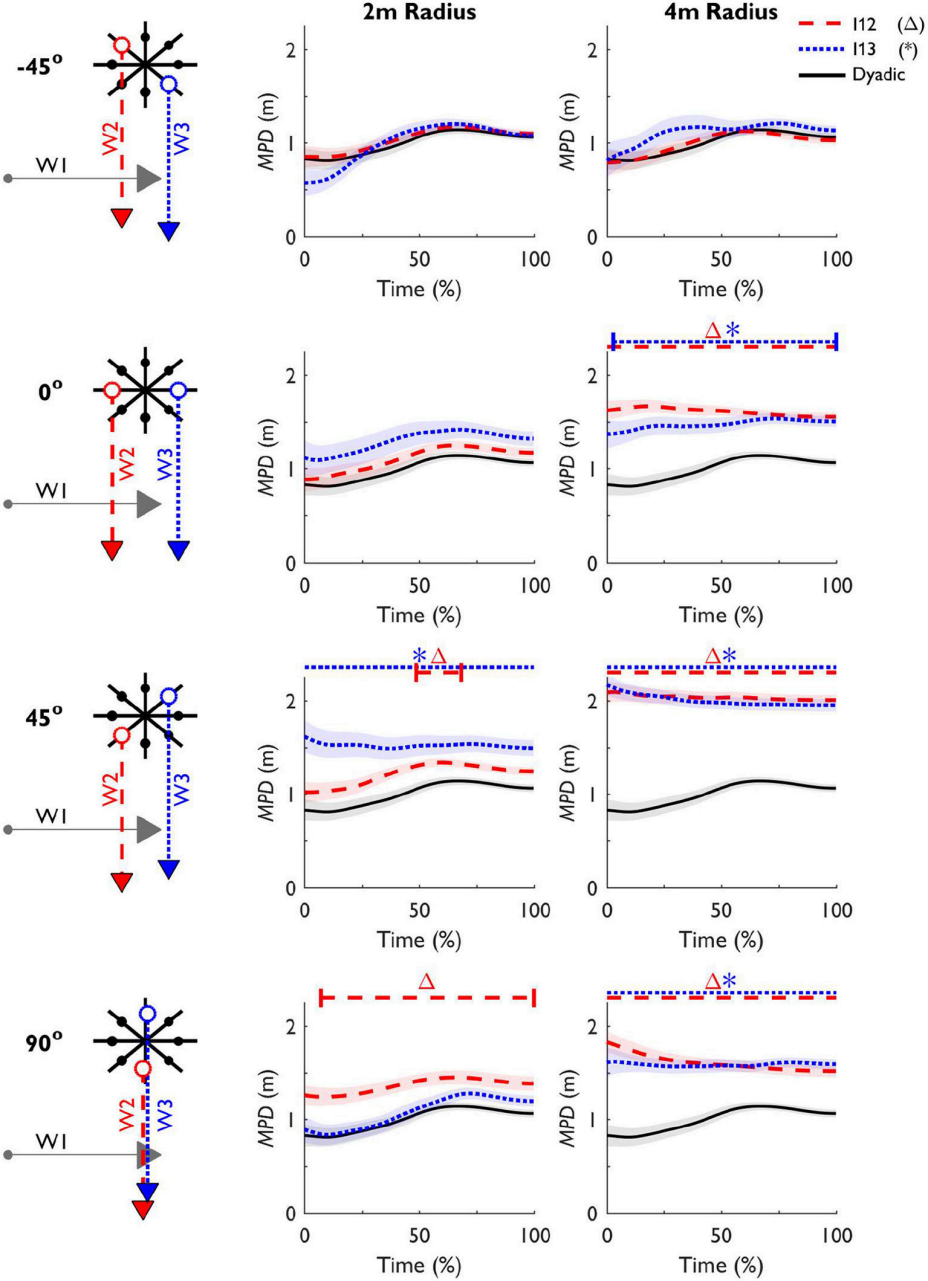
. Mean (±SE) the minimal predicted distance between Walker 1 & 2 and between Walker 1 & 3 (*MPD*_12_, dashed line; *MPD*_13_, dotted line, respectively) over time per formation. A horizontal line above the plots indicates when *MPD*_12_ (Δ) and *MPD*_13_ (*) are significantly different from the pairwise *MPD*_Dyadic_ (solid line) after a Bonferroni correction was applied. When the difference did not start at t_start_, small vertical bars were added to indicate first instant *MPD* was different from *MPD*_Dyadic_.

We examined the difference between the dyadic and triadic trials using SPM, comparing the normalized time-series of *MPD*_Dyadic_ with *MPD*_12_ and *MPD*_13_ in each formation separately (see Figure 5). All significant differences with a corrected critical *p*-value of 0.00625 are indicated in Figure 5 with a horizontal bar above the plot. Whenever the difference did not start at t_start_, short vertical bars were added to highlight the first instant a significant difference occurred. The SPM analysis revealed that in formation [90°, 2 m], *MPD*_12_ and in formation [0°, 4 m], *MPD*_13_ was significantly greater than the dyadic trials at t_end_, but not at t_start_ [*t*_(94)_ = 3.687, *p* = 0.002; *t*_(94)_ = 6.251, *p* = 0.001, respectively]. Additionally, this difference was already apparent from 7% onward in formation [90°, 2 m], and from 3% onwards in formation [0°, 4 m]. In formation [45°, 2 m], we also found a significant deviation of *MPD*_12_ from *MPD*_Dyadic_ *during* the trial. Between 49 and 68% *MPD*_12_ was significantly larger compared to *MPD*_Dyadic_ [*t*_(94)_ = 3.186, *p* = 0.003]. Furthermore, the SPM analysis revealed that *MPD*_12_ was significantly different from the dyadic trials from t_start_ until t_end_ in formation [0°, 4 m] [*t*_(94)_ = 8.043, *p* < 0.001], [45°, 4 m] [*t*_(94)_ = 13.632, *p* < 0.001], [90°, 4 m] [*t*_(94)_ = 6.875, *p* < 0.001]. Similarly, *MPD*_13_ was significantly different from the dyadic trials from t_start_ until t_end_ in formation [45°, 2 m] [*t*_(94)_ = 4.438, *p* < 0.001], [45°, 4 m] [*t*_(94)_ = 11.283, *p* < 0.001], [90°, 4 m] [*t*_(94)_ = 7.567, *p* < 0.001].

### Crossing order inversions

In the dyadic trials, 13% (6 out of 48) of the trials had an inversion of crossing order at some point during a trial. For the triadic interaction I12, inversions occurred in 12% (45 out of 383) of the trials. For I13, inversions occurred in 17% (65 out of 383) of the trials. The proportion of trials with inversions was not significantly different in the dyadic trials, I12 or I13: χ^2^(2, *N* = 814) = 4.401, *p* = 0.111.

### Gap pass-ability

**Figure 6:**
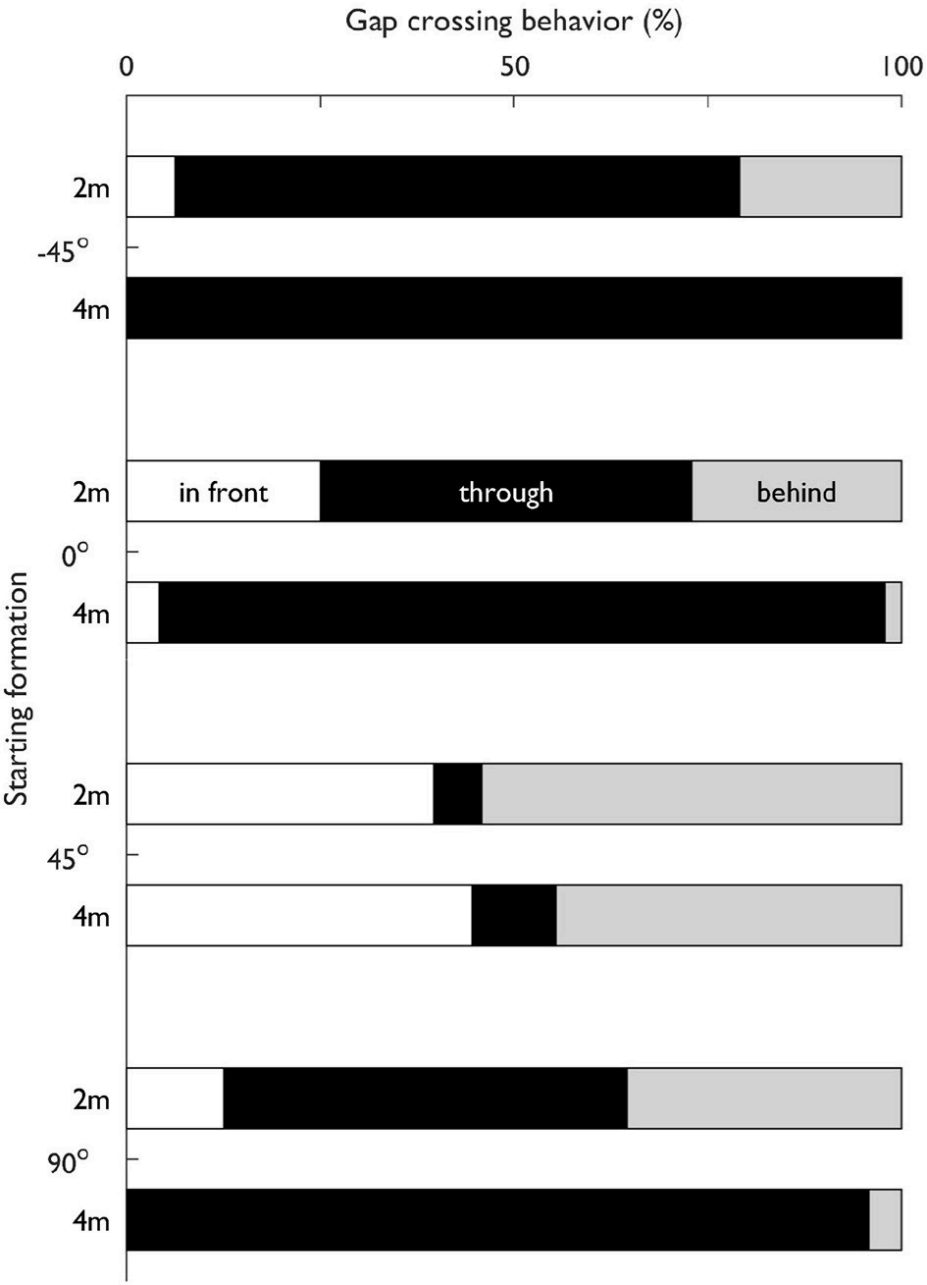
Gap crossing behavior (i.e., in front, through or behind) of Walker 1 (W1) relative to the other walkers in the triadic trials as a percentage per starting formation.

In Figure 6, the percentage of trials per formation with each gap crossing behavior (through or around) is shown. Formation significantly affected the gap crossing behavior, χ^2^ (14, *N* = 383) = 201.305, *p* < 0.001. In some formations W1 almost never passed through the gap ([45°, 2 m] and [45°, 4 m]), other formations allowed W1 to cross through the gap in almost all trials ([−45°, 4 m], [0°, 4 m], and [90°, 4 m]). It stands out that particularly when the radius was 4 m, W1 often crossed through the gap, except in formation [45°, 4 m].

**Figure 7:**
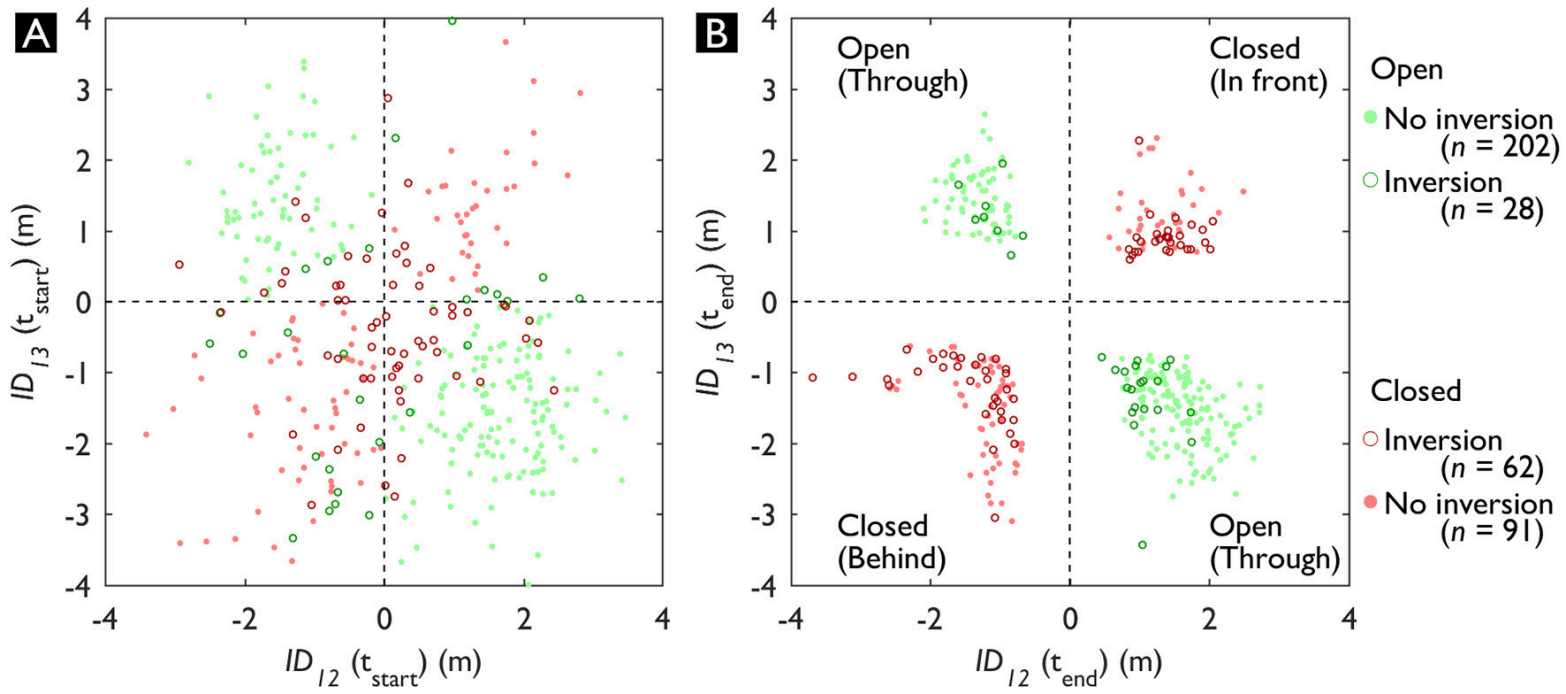
Distribution of the Interaction Distance (*ID*) in the triadic trials to both Walker 2 (W2) and Walker 3 (W3) at t_start_ **(A)** and t_end_ **(B)**. The colors indicate the final crossing order, the open circles indicate trials with a Dynamic Gap (*DG*) inversion. The dashed horizontal and vertical lines indicate the border between crossing in front and behind the other walker. When crossing behind both W2 and W3 (*ID*s < 0 m) or in front of both W2 and W3 (*ID*s > 0 m), the gap between them is closed for Walker 1 (W1). See also Video 2 of the Supplementary Material for an animated display from t_start_ until t_end_.

In Figure 7, *ID*_12_ and *ID*_13_ are visualized in relation to each other at t_start_ (Figure 7A) and t_end_ (Figure 7B). Figure 7A provides a descriptive insight in the distribution of the starting parameters of the triadic trials in terms of *ID* and therefore *DG*. Each quadrant represents the (predicted) state (open or closed) of the gap between W2 and W3, which is highlighted with the different colors. Moreover, the open circles and filled dots indicate whether for that trial an inversion occurred or not, respectively. For trials where W1 went through the gap (*n* = 230), an inversion of *DG* occurred in 12% of the trials. On the other hand, for trials where W1 went around the gap (*n* = 153), an inversion of *DG* occurred in 41% of the trials. That is, 12% of the green data points (see Figure 7B; top left and bottom right quadrant) and 41% of the red data points (see Figure 7B; top right and bottom left quadrant) were at some point in a differently colored quadrant (see Supplementary Material, Video 2). In the trials where W1 passed through the gap, *DG* inversions occurred significantly less often [χ^2^(2, *N* = 814) = 41.075, *p* < 0.001].

**Figure 8:**
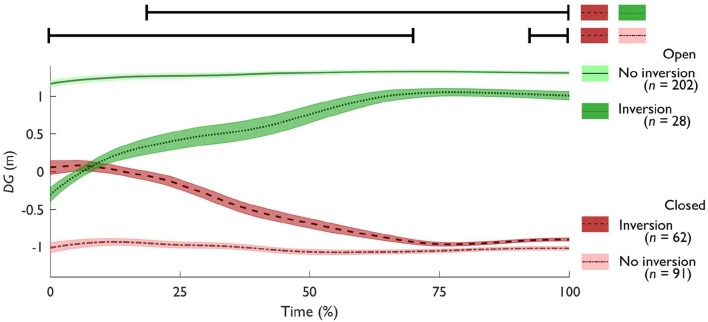
Average (±SE) Dynamic Gap (*DG*) over time in the triadic trials comparing through without (solid), and with inversion (dotted), around with (dashed), and without inversion (dash-dot). Positive *DG* values denote that the gap between Walker 2 and 3 (W2 and W3) is predicted to be open for Walker 1 (W1). Conversely, Negative *DG* values predict a closed gap. The *DG* value at t_end_ represents the gap crossing behavior. Each of these lines was significantly different from each other from t_start_ until t_end_; except for through and around with inversion, and for through with and without inversion. For the latter two, the significance bars above the figure indicate when the difference was significant.

For trials where W1 went through and around, with and without inversions, we separately plotted the average (±SE) values of *DG* for every time-step (see Figure 8). Using SPM, we compared each gap crossing behavior against one another (i.e., through and around, with and without inversion) with a corrected critical *p*-value of 0.0083. For trials with inversion, the trials where W1 went through the gap were significantly different from the trials where W1 went around the gap from 18% until t_end_ [*t*_(151)_ = 46.560, *p* < 0.001], as highlighted in Figure 8. For the trials where W1 went around the gap, the trials with inversion were significantly different from the trials without inversion from 0% until 70% [*t*_(151)_ = 13.582, *p* < 0.001] and later from 93% until t_end_ [*t*_(151)_ = 3.364, *p* = 0.004]. The remaining comparisons [around with inversion compared to through without inversion, *t*_(262)_ = 51.266, *p* < 0.001; through with compared to without inversion, *t*_(228)_ = 13.058, *p* < 0.001; through without inversion compared to around without inversion, *t*_(291)_ = 60.700, *p* < 0.001; through with inversion compared to around without inversion, *t*_(88)_ = 46.460, *p* < 0.001] were all significantly different from t_start_ until t_end_.

## Discussion

In the current paper, we compared dyadic (1 vs. 1) and triadic (1 vs. 2) interactions. We aimed to examine whether the extra walker in the triadic interactions changed how the interactions emerged. We manipulated the starting formation in the triadic interactions to obtain a range of initial parameters. We first assessed whether similar initial parameters in terms of *MPD* resulted in similar changes in *MPD* over time. Secondly, we compared whether the potential simultaneous treatment of the multiple interactions in the triadic trials resulted in a higher number of inversions. Lastly, we explored whether the triadic interactions could be described at the group level in terms of the pass-ability of the dynamic gap that may be formed between the two grouped walkers (W2 & W3). We found evidence that the same initial parameters resulted in different adaptations in the triadic compared to the dyadic trials. However, this potentially simultaneous avoidance strategy was not corroborated by a higher number of inversions. Furthermore, we successfully described how the potential gap between walkers can be linked to its pass-ability. Moreover, the pass-ability of the gap appeared to unfold through the bi-directional interactions and stabilize over time. We discuss the influence of the extra walker on the avoidance strategy in relation to the triadic formations and discuss the interactions in terms of the affordance of passing through the gap. Finally, we highlight how in some cases multiple interactions were possibly treated simultaneously.

### Dyadic vs. triadic interactions

We addressed our first research question - whether multiple interactions are treated sequentially - by examining the *MPD*. For some formations ([45°, 4 m] and [90°, 4 m]), the *MPD* was always significantly different from the dyadic trials. In these formations, both interactions could be negotiated without actively avoiding collision. When the risk of a collision is low, avoidance is not necessary (Olivier et al., 2012; Huang et al., 2017). It is thus uncertain whether the strategy in triadic interactions differed from the dyadic interactions. For the remaining formations, the same initial parameters (in terms of *MPD*) in the dyadic interactions did not always lead to the same avoidance behavior in the triadic interactions which we interpret in the context of simultaneous and sequential adaptations.

Out of the six comparable formations, three formations yielded a simultaneous collision avoidance strategy by W1. As evidenced by the difference in *MPD* at t_start_ in formation [90°, 2 m], the extra walker forced a quick and relatively large adaptation to secure a collision-free interaction. Once the collision risk of the first interaction was acceptable, the next interaction could be treated similarly to a dyadic interaction. In [0°, 4 m] on the other hand, the extra walker influenced the interaction, despite crossing at a large distance (i.e., with a low risk of collision). Moreover, in [45°, 2 m] the time-series analysis (see Figure 5) revealed a significantly higher *MPD* halfway the interaction compared to the dyadic interactions. It can be argued that the extra walker resulted in these augmented responses, reflecting a degree of simultaneous treatment of the interactions. Similarly, Bruneau et al. (2015) showed that depending on social constraints such as group density and appearance, walkers might prefer to avoid having to interact with members of a group individually by going around a group as a whole. Such simultaneous treatment of the interactions could be a conservative strategy to simplify interacting with multiple walkers, which is a common strategy when uncertainty increases (Krell and Patla, 2002; Berard and Vallis, 2006; Lowrey et al., 2007).

Finally, some triadic trials showed no difference with the dyadic trials during the interaction ([−45°, 2 m], [−45°, 4 m], and [0°, 2 m]). In terms of the evolution of *MPD*, this indicates that the triadic interactions were negotiated as sequential dyadic interactions. It remains somewhat ambiguous whether these interactions were treated sequentially, as perhaps this was a result of the convenient positioning of the extra walker. The symmetrical set-up of the triadic formation allowed for W1 to go in front of one, and behind the other walker, with acceptable risks of collision. Indeed, formations [−45°, 2 m], [−45°, 4 m], and [0°, 2 m] had a high incidence of going through (see Figure 6). Overall, it can be argued that, when the extra walker is conveniently positioned, triadic interactions can be negotiated with sequential dyadic interactions. However, the extra walker can also interfere with the original strategy, hence resulting in a strategy where W2 and W3 are avoided simultaneously. These findings highlight that a good understanding of the micro-level interactions that construe crowd movements requires a close examination of how multiple interactions are engaged.

Previously, preservation of crossing order has been reported as one of the rigid characteristics of pairwise (i.e., dyadic) locomotor collision avoidance (Olivier et al., 2013; Knorr et al., 2016; Lynch et al., 2017). Therefore, we addressed our second research question in terms of crossing order inversion. Interestingly, we found a high rate of crossing order inversion compared to previous research, even in the dyadic trials (13%). In the triadic trials, inversions occurred in 12% and 17% in I12 and I13, respectively, but in contrast to our hypothesis inversions did not occur significantly more often in the triadic compared to the dyadic interactions. One difference with the previous studies by Olivier et al. (2012, 2013) is that in the current study participants could already see each other at the starting positions (similar to Basili et al., 2013; Huber et al., 2014). Early visual information could influence the interaction, particularly given that early adaptations have a bigger effect on the crossing distance. Inversions typically indicate that there was some asymmetry in the interaction, that is, one pedestrian contributed more to the change compared to the other. Vassallo et al. (2017) showed for example that in a human-robot interaction, humans prefer to let the robot cross first, even if that means inverting the crossing order. These asymmetries could be considered in the context of social motor coordination (cf., Schmidt et al., 2011). For example, Dicks et al. (2016) reported that the collision avoidance strategy depends on the potential for social interaction, which was manipulated by having oncoming walkers look at their mobile phones or not. In the absence of gaze, pedestrians adjust their strategy (Croft and Panchuk, 2017), arguably to anticipate that the other walker may not initiate any adaptation (cf., asymmetrical coupling in interpersonal coordination; Meerhoff and de Poel, 2014). This social component can also be modeled mathematically. For example, Colombi and Scianna (2017) made a first attempt to include the subjective perception of the attractor-state of multiple persons in their model. Although the model did not expand on an agent's action opportunities, it did report the that subjective perception influenced sequential (i.e., localized perception) and simultaneous (i.e., distributed perception) interactions.

### Pass-ability

For our third research question, we assessed the pass-ability of the gap between W2 and W3 with respect to W1. Looking at the triadic interactions, the gap crossing behavior was clearly affected by formation (see Figure 6). In some formations ([45°, 2 m] and [45°, 4 m]), W1 almost never passed through the gap, other formations allowed W1 to cross through the gap in almost all trials ([−45°, 0°] and [90° at 4 m]). It stands out that particularly with a 4 m radius, W1 often crossed through the gap, except at −45°. This indicates that participants were susceptible to the affordance of passing through a gap between others (Plumert and Kearney, 2014). Inversions were less frequent in trials where W1 ended up going through the gap (12%) compared to W1 going around the gap (41%). In other words, gaps that were initially predicted to be open ended up being closed more often than vice versa. This could indicate that going around was more inviting (Withagen et al., 2017) compared to going through. In other words, the attractor-state of going around was more stable (Schmidt and Richardson, 2008). In contrast to our hypothesis, the initial parameters in terms of *DG* did not result in less inversions compared to the initial parameters in terms of *MPD*, with inversions only occurring in 12–17% of the trials. This highlights how low the prediction accuracy of gap crossing behavior is at t_start_. When subsequently examining the time-evolution of *DG*, it becomes clear that only after 18% of time the predicted crossing behavior on average matched the final gap crossing behavior. It may well be that the triadic interactions were inherently more dynamic because of the mutual (i.e., bi-directional) interactions, which has previously been observed even in a highly controlled setting (Meerhoff and de Poel, 2014). As can be seen in Figure 8 and Video 2 of the Supplementary Material, the predicted outcome in triadic trials can change until quite late in the interaction. Therefore, rather than looking at the initial parameters, the dynamics of a gap need to be considered to examine the reciprocal interactions between all walkers throughout an interaction using a descriptive variable such as *DG*. Our *DG* jointly captures the behavior of three walkers in terms of their speed and heading. which can shed light onto how the gap crossing behavior unfolds. However, one could argue that to classify a gap as pass-able, it is pertinent to consider the space necessary to pass through a gap. Previous work has highlighted how the pass-ability of a gap is tightly coupled to the shoulder to aperture ratio (e.g., Wilmut and Barnett, 2010; Franchak et al., 2012; Hackney et al., 2015). With *DG*, we have taken the first hurdle toward assessing multiple pedestrian interactions at the micro-level by quantifying the magnitude of a potentially pass-able gap. Future work could extend this measure by looking at how the magnitude of *DG* and the final gap crossing behavior relates to body-scaled characteristics such as the shoulder to aperture ratio. In addition to examining I12 and I13, future work could for example use gaze (Meerhoff et al., 2018) to also focus on I23 - the interaction between the grouped walkers - to tease apart whether pedestrians in a crowd form a coordinated strategy to let others pass between them. Depending on how the interactions between W2 & W3 (i.e., within a group) are perceived, the decision to go through or around are strongly affected (Bruneau et al., 2015). If W2 and W3 are perceived as a coordinated unit, it might be less inviting to go through a potential gap between two walkers.

The different gap crossing behaviors (through or around, and with or without *DG* inversions) may reveal an extent of sequential or simultaneous treatment of the interactions by W1. It could be argued that trials without *DG* inversion allowed for simultaneous treatment of the interactions, as the predicted crossing behavior corresponded with the final gap crossing behavior. Trials with *DG* inversions could be interpreted as sequential: First, W1 and W2 interacted, and then W1 and W3. This is perhaps similar to the *MPD* differences in formation [90°, 2 m], where I12 first reached an “acceptable” risk of collision to subsequently continue treating I13 as a dyadic interaction. However, similar to the *MPD* differences between dyadic and triadic trials (see above, [−45°, 2 m], [−45°, 4 m] and [0°, 2 m]), it remains ambiguous whether these interactions were treated simultaneously intentionally, or whether this was simply due to the convenient positioning of W2 and W3. It is difficult to say whether some formations simply did not afford sequential treatment, or whether the simultaneous treatment was in fact coincidental. More work is required to better understand to what extent interactions can be treated simultaneously. Current models of understanding how walkers combine multiple interactions are typically based on an arbitrary selection procedure, for example based on whether the other walkers are within view and within a critical distance (e.g., Helbing et al., 2001). However, human-to-human interactions seem to be governed by subtler behavioral laws. Despite the relatively few unique participants, our results showed that these interpersonal dynamics are highly adaptive and not exclusively restricted by a critical distance. The generalizability of this finding can be strengthened by adopting a random effects model (e.g., Barr et al., 2013), which more appropriately deals with the between-subject variability. Nevertheless, it can be surmised that the adaptive human-to-human behavior needs to be taken into account for understanding the micro-level interactions. However, more work is required to assess what makes the pass-ability of a gap attractive enough to act upon it.

Another direction for future work could be to expand on how this behavior may be guided visually. As for example highlighted by Zhao and Warren (2017), on-line visual information is pertinent for locomotor interception of a moving target. Moreover, Dachner and Warren (2016) explain how depending on a person's location (in front or to the side), pedestrian following can be explained by a combination of a target's bearing and optical expansion. Both locomotor interception and pedestrian following are important components of pedestrians navigating through crowds of people. For example, it could be that the bearing angle provides pertinent information to avoid collision with multiple persons. However, Chihak et al. (2010) showed that when cyclists cross through gaps in traffic, humans do not simply adjust their action to the bearing angle. Although the initial strategy seems consistent with a bearing angle strategy, they observed a multi-stage strategy as a cyclist got closer to the actual gap. Nevertheless, it may be that some form of the bearing angle (e.g., a fractional order, see Bootsma et al., 2015) or another optical variable could indeed explain how collision with multiple walkers is guided visually. However, in the case of avoidance, the rate of change of bearing angle becomes infinite at the instant of smallest distance, which makes it difficult to apply it to this specific situation. As an alternative, we propose that navigation through a crowd of people can be considered as passing through a multitude of dynamic gaps. Watson et al. (2011) provide a good basis for determining how pass-ability of a gap can be optically specified. In their study, they assessed the perception of whether a gap between two rugby defenders affords passing a ball through. They showed that 82% of the variance could be accounted for based on tau-based information (Lee, 1976, 1998). Future work could examine which optical variables may specify the pass-ability of a dynamic gap between pedestrians.

## Conclusion

We showed that triadic (1 vs. 2) interactions are not always comparable with dyadic (1 vs. 1) interactions. Although it can be argued that a conveniently positioned extra walker allowed for similar triadic compared to dyadic interactions, the extra walker can also interfere with the original strategy, hence resulting in a different adaptation. Even more than dyadic interactions, triadic interactions strongly depend on how the reciprocal interactions between all walkers unfold. Moreover, the interpersonal dynamics are highly adaptive and not (only) restricted by a critical distance. We adopted a novel analysis to describe this dynamic character of the gap between two walkers. By describing the affordance of passing through a gap *over time*, the emergence of the final gap crossing behavior can be better understood. Furthermore, we propose that some interactions afforded simultaneous treatment and others required a more sequential treatment of the interactions. This study was a first attempt to understand the combination of multiple interactions at the micro level. However, future research should specifically address under which circumstances these different types of interactions occur. For example, using a virtual reality environment, the trajectories of the interfering walkers can be controlled to design a paradigm that contrasts simultaneous and sequential treatments of interactions. In sum, we revealed that in some cases, triadic and dyadic interactions yield different collision avoidance strategies and interestingly, the interactions between multiple persons unfold over time through bi-directional interactions.

## Author contributions

LM, JP, AC, and A-HO conceived and planned the experiments. LM, JP, AC, and A-HO carried out the experiments. LM took the lead in the data analysis and writing of the manuscript. LM, SL, JP, and A-HO provided critical feedback and helped shape the research, analysis and manuscript.

### Conflict of interest statement

The authors declare that the research was conducted in the absence of any commercial or financial relationships that could be construed as a potential conflict of interest.

## Footnotes

^1^The code for the kinematic analyses can be found in the supplementary material and on https://github.com/Rens88/PW_to_Multiple_Public. The ‘exampleRun.m’ can be used to run the code with some mock-data. Data can be made available upon request.

^2^SPM (Statistical Parametric Mapping) is a commonly used open source statistical tool in neuroimaging (available on http://www.spm1d.org), specifically, through the application of Random Field Theory (RFT) that makes inferences of topological brain image features that are continuous functions of space or time (Adler, 1981; Pataky, 2010, 2016). The methods are based on a General Linear Model (GLM) of analysis; in its simplest expression, SPM runs an analysis per time point, grouped as a continuous statistical process and tested for probabilistic behavior through RFT using univariate probability and spatial covariance. The probabilities are computed from expected Gaussian random fields (Adler and Hasofer, 1976), which in turn leads to probabilistic descriptions of distribution that in turn allow for common parametric analyses such as GLM and ANOVA. See for a more detailed explanation Pataky (2010) section 2; and for full technical details Pataky (2016). Of the software package, we specifically used the paired comparison in spm1d (spm1d_stats_ttest2.m) and our inferences were based on a GLM (spm1d_stats_glm.m).
